# Fabrication and Characterization of Au Nanoparticle-aggregated Nanowires by Using Nanomeniscus-induced Colloidal Stacking Method

**DOI:** 10.1007/s40820-014-0015-3

**Published:** 2014-10-25

**Authors:** Sangmin An, Wonho Jhe

**Affiliations:** 1 Department of Physics and Astronomy, Institute of Applied Physics, Seoul National University, Daehak-dong, Gwanak-gu, Seoul, 151-747 South Korea; 2 Present Address: Center for Nanoscale Science and Technology, National Institute of Standards and Technology, Gaithersburg, MD 20899 USA

**Keywords:** Au nanoparticle-aggregated nanowire, Nanomeniscus-induced colloidal stacking method, Atomic force microscope, Liquid–solid coexistence phase

## Abstract

We fabricate and characterize Au nanoparticle-aggregated nanowires by using the nano meniscus-induced colloidal stacking method. The Au nanoparticle solution ejects with guidance of nanopipette/quartz tuning fork-based atomic force microscope in ambient conditions, and the stacking particles form Au nanoparticle-aggregated nanowire while the nozzle retracts from the surface. Their mechanical properties with relatively low elastic modulus are in situ investigated by using the same apparatus.

## Introduction

The characteristics of the nanowires (NWs), which are one of the critical bridging elements in nanoscience and technology, have been widely studied. In particular, special attention has been focused on various applications, such as biomedical sensing, nano-optoelectronics, and photovoltaic devices due to their advanced electrical, optical, mechanical, and geometrical properties [1–5]. Since the vapor–liquid–solid fabrication method of NW was first invented [6], based on the crystal seed-based growth method, the research of NW has been established as a convergence field [7–9]. Recently, various materials of NW composite have been investigated for versatile functionalization in specific systems and devices [10, 11]. These fabricated NWs are mostly crystalline solid phase and thus have important features of high-speed operation and reproducible response suitable for the nanodevices and related technologies. However, unlike the solid-state applications, nanoscale biological systems are involved with objects that usually exhibit soft matter properties, such as the liquid–solid coexistence (LSC) phase which can be defined by the volume fraction of the constituent particles in the liquid [12, 13]. Thus, the fabrication and characterization of soft NWs gives a key for understanding complicated biological systems. Moreover, investigation of their physical properties consequentially should be performed for the one dimensional applications of bioscience and technology. Here, we introduce a direct non-template fabrication and characterization of the Au nanoparticle-aggregated (ANA) NWs which shows soft matter properties using nanomeniscus-induced colloidal stacking method in ambient conditions. We used a non-contact, small lateral oscillation mode, nanopipette [14] combined with a quartz tuning fork-atomic force microscope (QTF-AFM) [15] for Au nanoparticle solution delivery, which is one of tool for scanning probe lithography [16]. And we in situ investigated the mechanical properties of ANA-NWs which show a relatively low elastic modulus by using the same apparatus facilitated with QTF sensor for small force measurement.

## Experimental

**Fig. 1:**
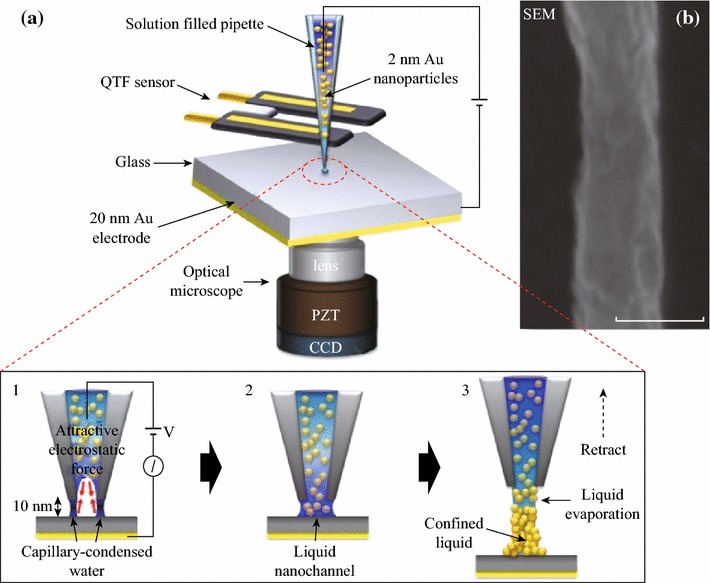
Proposed system and result. **a** Schematic of fabrication process of Au nanoparticle-aggregated (ANA) nanowires (NWs) with colloidal stacking method. (*1*) When the nanopipette tip approaches the substrate, the nanomeniscus-induced ejection of the 2 nm Au particle solutions occurs at a low voltage bias (~10 V) in ambient conditions (*2*) with formation of liquid nanochannel. (*3*) The colloidal stacked ANA-NW forms as the tip retracts in 3D space while the liquid solution evaporates. **b** SEM image of the 100 nm (*scale bar*) diameter ANA-NW

**Fig. 2:**
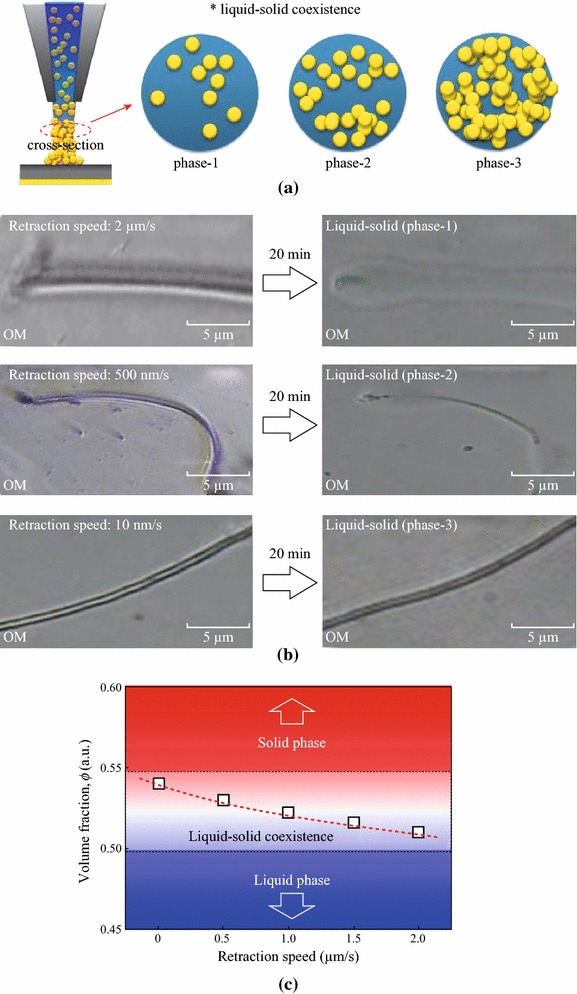
Liquid-solid coexistence (LSC) phase of the ANA-NW. **a** Schematic of phase-1 ~-3. **b** Dependence of the retraction speed. 20 min later each case of retraction speed (10 nm/s, 500 nm/s, 2 μm/s) phase shows different behaviors, such as entire melting (phase-1), local melting (phase-2), no melting (phase-3). **c** Predicted phase map of the ANA-NWs

**Fig. 3:**
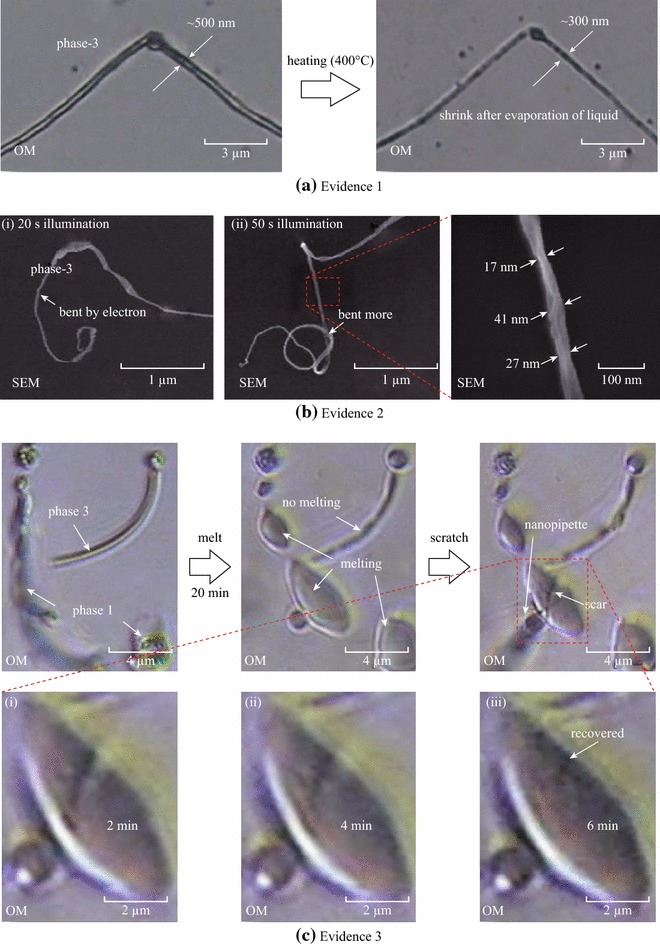
Physical properties of ANA-NWs. **a** Shrinking: Heating test results shrinking of NW’s diameter. **b** Bending: The NW bends and shrinks by electron in the SEM environment. **c** Recovery: The NW of phase-1 is scratched and got a scar with the same nanopipette tip, and the scar is completely recovered after 6 min later

Figure 1a shows schematic of fabrication process of ANA-NW using nanopipette-combined QTF-AFM. The pulled nanopipette tip made by mechanical puller (P-2000, Sutter Instrument, Co.) is filled with a commercial 2 nm Au nanoparticle solution (2 ± 0.2 nm diameter, PBS buffer, 0.01 % wt/vol concentration, BBI Solution Co.), and then attached on the side of the QTF sensor’s prong for approaching the surface with atomic resolution. After the tip approaches the surface within 10 nm, the capillary-condensed nanoscale water meniscus [17] was formed between the apex of the pulled pipette and the surface. And the liquid solution was ejected onto the surface via attractive electrostatic force which exerts between the condensed water meniscus and inside solution (Fig. 1a-1). This forms liquid nanochannel for continuous flow (Fig. 1a-2). Then the pipette tip was immediately retracted to the opposite direction of the surface while the inside solution continues to extrude and forms a colloidal stacked ANA-NW with evaporation of the liquid (Fig. 1a-3). Figure 1b shows the SEM images of the fabricated ANA-NW with ~100 nm diameter which are determined by aperture size of the pulled nanopipette. The length can be easily controlled by *z*-axis retraction movement of piezoelectric transducer (PZT), and the softness is determined by tip retraction speed with respect to stacked colloidal density.

## Results and Discussion

### Control of Stacked Colloidal Density

The phase of the fabricated ANA-NWs shows behaviors of LSC phase such as melting with the different volume fraction according to control parameter of the retraction speed of *z*-axis observation of the three phases of LSC for the fabricated NWs, which are determined by the retraction speed of nanopipette tip movement. Figure 2 presents the definition, and experimental (equivalent to the NW growth rate) and the resulting volume fraction. In Fig. 2a, the cross-sectional composition of the NWs defines the liquid-like phase-1, intermediate phase-2, and solid-like phase-3, depending on the density of the Au nanoparticles dispersed in the liquid solvent. Figure 2b shows the OM images of the NWs fabricated at three different tip retraction speeds (2 μm/s, 500 nm/s, and 10 nm/s), where fast (slow) pulling produces phase-1 (phase-3). Although the pulled liquid preform in the form of nanobridge determines the initial shapes of the NWs, they change with time as evaporation of the liquid (i.e., deionized water and solvent) progresses. The respective NW shapes observed after 20 min elapse show very differing behavior; liquid-like melting (phase-1), partial melting (phase-2), and solid-like hardness (phase-3). The results can be attributed to the fact that slow pulling by slow tip retraction allows sufficient time for compact stacking of the nanoparticles before the liquid evaporates [18, 19], whereas melting [20, 21] dominates when fast pulling leaves a low constituent particle density. Figure 2c shows the predicted LSC phase map of the NWs, which plots the volume fraction versus the pulling speed, obtained by in situ observation of the melting speed and melted fraction. The volume fraction value of the fabricated LSC phase of ANA-NW may locate between the values of liquid and solid phase. The defined volume fractions (freezing 49.4 % and melting 54.5 %) at two interfaces is described from the criteria of the sedimentation of crystals in hard-sphere, monodisperse colloidal suspensions, which values are for the understanding guidance and could be varied by the selected materials [22]. One can calculate accurately the volume fraction by counting the resulting number distribution of individual nanoparticles using confocal microscope image analysis accompanied by molecular dynamics simulation [23].

### Physical Properties of ANA-NWs

Figure 3 discusses three physical properties of the LSC phases of NWs. Figure 3a shows shrinking due to liquid evaporation by heating the phase-3 NW up to ~400 °C, whose diameter shrinks by nearly 40 % due to liquid (solvent) evaporation. The second is that the NW bends and shrinks via electron illumination (Fig. 3b). The SEM images of the bent NW were taken immediately after electron-exposure time of (i) 20 s and (ii) 50 s. Notice that the random nature of liquid evaporation results in the different shrunk diameters (17, 27, and 41 nm) for 50 s long exposure. Figure 3c presents the recovery of LSC phase-1, which is monitored by in situ OM observation on the surface. After two types of NWs (phase-1 and -3) are fabricated on the surface using the parameter (retraction speed: 2 μm/s) shown in the phase map of Fig. 2c, we waited for 20 min and found only the phase-1 NW melted down, which is consistent with the phase-1 result in Fig. 2b. In addition, the phase-1 NW was intentionally scratched by the sliding nanopipette tip in contact with the surface, which left a line scar in the middle of the melted NW. Then, we observed that the scar gradually disappeared and liquidity of phase-1 was recovered in a slow recovery time of about 6 min like jelly or butter (Fig. 3c-i–iii).

### In Situ Measured Shear Modulus of ANA-NWs

**Fig. 4:**
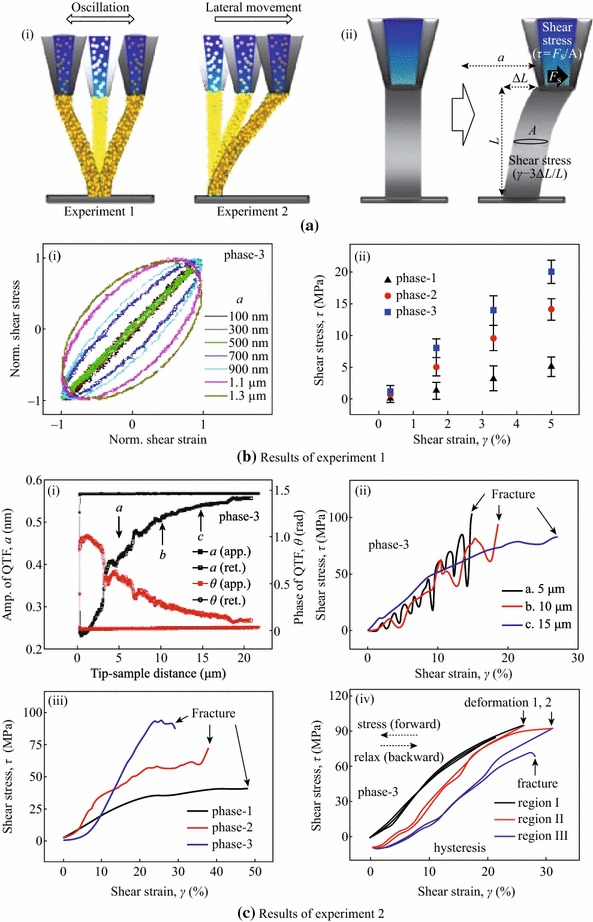
Mechanical properties of the fabricated ANA-NW. **a** (*i*) Schematics of two measurement methods for the NWs; fast oscillation motion by the QTF tip (experiment 1) and slow lateral movement by the PZT (experiment 2). (*ii*) The resulting strain–stress analysis provides mechanical properties including the shear modulus. **b** Results of the fast oscillation (at ~32 kHz frequency) experiment. (*i*) Hysteretic responses between the shear stress and strain for a different oscillation amplitude a. (*ii*) Value of shear modulus of phase-1 is higher than phase-3 case. **c** Results of the slow lateral movement (at 50 nm/s speed) experiment. (*i*) The QTF responses during approach and retraction of the tip, which can be used to control fabrication of variable-length NWs. (*ii*), (*iii*) The stress–strain curves for different displacements and LSC phases, respectively. The slope measures the similar value of shear modulus; ~400 MPa for the phase-3 NW. (*iv*) Stepwise repetition of the stress and relax on the NW shows the deformation (region I) followed by the degraded hysteresis behavior (region II), which eventually leads to fracture (region III)

We have investigated the mechanical properties of the target materials by interpretation of the pre-installed QTF force sensor with model of simple harmonic oscillator [24–26]. When the ANA-NW was formed between the nanopipette tip and the substrate, with its bottom end fixed to the substrate via physical bonding, two kinds of experiments were performed for the nanofiber; fast oscillation motion by the QTF tip (experiment 1) and slow lateral movement by the PZT (experiment 2). Figures 4a-i and ii show the schematics of the two experiments and the corresponding stress and strain analysis, respectively. The shear modulus (=stress/strain, *G* = *τ/γ*) of the NWs can be measured in both experiments 1 and 2, where the shear stress and strain are given by *τ* = *F*_*s*_/*A* and *γ* = 3 Δ*L*/*L*, respectively. Here, the shear force *F*_*s*_ is obtained by integration of the QTF sensor’s force-gradient results, *A* is the circular cross-sectional area of the NW, lateral displacement Δ*L* is half of the oscillation amplitude *a* of the QTF, *L* is defined as the stopping position of tip retraction from surface, and the factor 3 comes from the cantilever-model approximation.

Figure 4b shows the results of the fast oscillation experiment 1 for the phase-3 NW with ~15 μm length and ~100 nm in diameter. The oscillation frequency of the NW is same as the resonance frequency of the QTF (~32 kHz), while each lateral displacement Δ*L* is half of the oscillation amplitude *a* of the QTF (100 nm–1.3 μm), determined by the stroboscope images of QTF sensor [27]. Figure 4b-i presents the elliptic hysteresis curves of the stress and strain, which reveal the time delay Δ*t* between stress and strain, that is, the viscoelasticity information of the NWs. As *a* (or Δ*L*) is increased, the normalized hysteresis curves exhibit increase of the enclosed area of the ellipsoid, which indicates the strain energy per unit volume that is released as internal heat in each cycle. For large oscillation (*a* = 1.3 μm), the measured Δ*t* is about 5 μs, whereas only a slight delay occurs for small oscillation (*a* = 100 nm), indicating the dependency of viscoelasticity of the phase-3 NW on the oscillation velocity. Figure 4b-ii plots the shear stress versus the strain for three different phases of the NW and the slopes represent the shear moduli of the phase-1, -2, and -3 NWs given by ~100, 220, and 400 MPa, respectively. The modulus of phase-1 of the NW is about 4 times smaller than the results of phase-3, namely, the shear modulus of the ANA-NW increases, as the density of the particles increases.

Figure 4c studies the lateral movement (experiment 2) of the same 15 μm-long NW, while the PZT provides slow (at ~50 nm/s) unilateral displacement Δ*L*, where the QTF is now used as a force sensor. Figure 4c-i presents the QTF sensor responses versus the tip-sample distance during approach and retraction of the sample, which shows that the NWs can be fabricated at an arbitrary length, as marked by three arrows (a, b, and c) (here, liquid ejection starts at the zero-distance position). Figure 4c-ii plots the dependence of the NW’s length on the stress–strain curves. As the length increases from a (5 μm) to c (15 μm), the fracture occurs at the increased shear strain, whereas the shear stress at the fracture slightly decreases by ~20 MPa. The fluctuating behavior of the shorter NWs (5 and 10 μm) is attributed to rearrangement of the constituent nanoparticles as the NW is laterally stressed, which is averaged out in the longer 15 μm NW over its length probably due to its lesser sensitivity to movement of individual nanoparticles. In Fig. 4c-iii, the differing shear stress–strain curves are presented for the three LSC phases, where each slope provides the shear modulus of about 80, 180, and 400 MPa, respectively. The good agreement of the values with those of experiment 1 (Fig. 4b-ii) indicates that the oscillation motion at 32 kHz is still slow enough to be considered as static. Note that the measured shear modulus of the phase-3 NW shows similar values with the protein crystal (100–1,000 MPa) or ANA-polymer (~500 MPa) [28]. Figure 4c-iv shows the repetitive stress–strain measurements for the phase-3 NW during stepwise increase of the shear strain followed by the subsequent relaxation. Up to ~25 % strain (black curve, region I), there is no significant hysteresis between the forward and backward displacements. However, at above ~25 % strain, permanent deformation (or thinning) of the NW occurs, accompanied by a sudden slight drop of the shear stress and recovery of zero strain (red curve, region II). Then, a second deformation takes place at ~30 % strain, followed by another hysteresis and subsequent ultimate fracture.

## Conclusion

We have fabricated and characterized the ANA-NW with LSC phase by using colloidal stacking method with a guidance of nanopipette/QTF-AFM which operates in a non-contact, small lateral oscillation mode in ambient conditions. One can progress scientific improvements dealt with vital phenomena of low dimensional biological media using this fabricated LSC phase ANA-NW, 3D nanoscale structures of particle-aggregated system with various materials (inks) for electrical/biological/chemical engineering, or development of molecular electronics.
